# Polymers of functionalized diaminopropionic acid are efficient mediators of active exogenous enzyme delivery into cells

**DOI:** 10.1038/s41598-024-64187-1

**Published:** 2024-06-08

**Authors:** A. Romanowska, P. Rachubik, A. Piwkowska, M. Wysocka

**Affiliations:** 1 Faculty of Chemistry, University of Gdansk, Wita Stwosza 63, 80-309 Gdańsk, Poland; 2 Laboratory of Molecular and Cellular Nephrology, Mossakowski Medical Research Institute Polish Academy of Sciences, University of Gdansk, 80-308 Gdańsk, Poland

**Keywords:** Cationic peptidomimetic, DAPEG polymer, In-cell protein delivery, Biotechnology, Chemical biology, Chemistry, Polymer chemistry, Bioconjugate chemistry

## Abstract

Delivery of active protein especially enzyme is one of the major therapeutic challenge. Replacing or substituted invalid/improper acting protein offer fast and effective treatment of disease. Herein, we describe the synthesis and properties of biotinylated peptidomimetics consisting of oxoacid—modified 2,3, l-diaminopropionic acid residues with guanidine groups on its side chains. Electrophoretic analysis showed that the obtained compounds interact with FITC-labeled streptavidin or a streptavidin-β-galactosidase hybrid in an efficient manner. Complexes formed by the abovementioned molecules are able to cross the cell membranes of cancer or healthy cells and show promising compatibility with live cells. Analysis of β-galactosidase activity inside the cells revealed surprisingly high levels of active enzyme in complex-treated cells compared to controls. This observation was confirmed by immunochemical studies in which the presence of β-galactosidase was detected in the membrane and vesicles of the cells.

## Introduction

Delivering enzymatically active proteins to the cell interior is one therapeutic approach that can be used to restore homeostasis inside the cell. Recent examples of such approach are caspases 8 or phosphatase 1B^1,2^. Numerous delivery approaches allow the translocation of active proteins, including viral and nonviral carriers, nanoparticles, and lipid-based particles^3^. Systems that are able to deliver cytosolic protein would offer new approaches for combatting a variety of diseases, including cancer^4^, diabetes^5^, inflammation^6^, and neurodegenerative disorders^7^. Exogenous or nonhost protein-coding DNA or RNA sequence introduced into a cell or organism can be translated but may lead to a misfolded or improperly post translationally modified final product. Thus, delivery of active native proteins could be an interesting alternative to the abovementioned approaches. Two factors are crucial to maintain during the delivery process: an effective concentration and the function of the translocated protein. Among the nonviral vectors, several covalent and noncovalent systems for model protein translocation have been described to date.

One of most effective seems to be the synthesis of cell-penetrating peptide (CPP)-conjugated protein hybrids^8^. The authors of this study utilized a polyarginine (poly-Arg) sequence. Poly-Arg sequence has also been used to modify the surface of nanoparticles or to form noncovalent complexes with selected cargo proteins.

However, it should be noted that Arg-rich peptides display moderate cytotoxic effects toward some eukaryotic cells, which limits their broad application^9,10^. This important topic has been extensively and superbly reviewed by Lee et al.^11^. Recently, we developed a series of compounds being polymers decorated by guanidine groups that are able to promote DNA condensation and mediate gene delivery in a cell-safe manner^12,13^. Using this approach, we were able to induce GFP expression in cancer and healthy cell lines^14^.

Delivery of β-galactosidase (further abbreviated as β-gal) as a model enzyme has been utilized by several groups using different compounds, including noncovalent complexes formed by excess CPP pep-1^15^ or covalent attachment of numerous molecules of the nucleus-targeting peptide NrTP6^16^.

**Figure 1:**
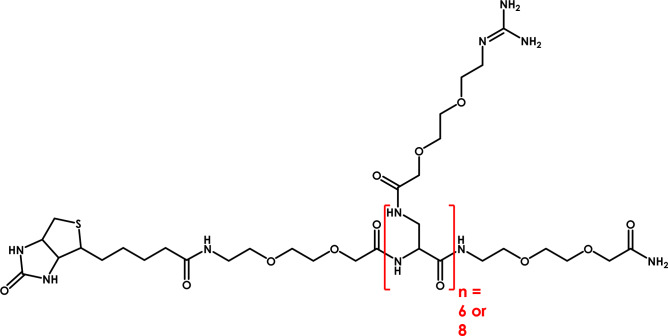
General scheme of the synthesized compounds.

In the present work, we describe the use of biotin-labeled molecules, whose structures were shown on Fig. 1 and described above, in an effective protein translocation model. To investigate the efficacy of this process, we used two different proteins: FITC-streptavidin and streptavidin-β-gal hybrid protein. All proteins were expressed in an *E. coli* system.

Our preliminary data obtained for a series of healthy breast and breast cancer cell lines indicate enrichment of β-gal and, more importantly, its activity within the treated cells. Such novel approach could be interesting alternative for current therapeutic methods.

## Materials and methods

### Synthesis

The peptidomimetics were synthesized according to a procedure described previously by our team^14^. The biotin moiety was attached to the N-terminal amino group using a molar excess of Bt-NHS:DIPEA (3:1.5) in NMP. The completeness of the reaction was monitored by the Kaiser test.

### Cell lines

The healthy cell line HB2 (human breast epithelial cells) was obtained from Merck (Germany), the cancer cell line MDA-MB-231 (human breast cancer epithelial cells) was obtained from ATCC (United Kingdom), the cancer cell line SKBR3 (human mammary gland cancer) was obtained from CLS (Germany), and the cancer cell line T47D (human breast cancer epithelial cells) was obtained from CLS (Germany). All cell lines were cultured at 37 °C in 5% CO_2_ in Dulbecco’s modified Eagle medium (DMEM) supplemented with 4.5 g/L glucose, 10% fetal bovine serum (FBS) and 1% penicillin‒streptomycin solution (100 units of penicillin and 100 µg/mL streptomycin). The HB2 cell line requires the addition of 5 µg/mL insulin and 5 µg/mL alcoholic hydrocortisone solution.

### Complex formation and electrophoresis assay

#### FITC-Strp: Bt-O2Oc-[Dap(GO2)]_6_-O2Oc-NH_2_/Bt-O2Oc-[Dap(GO2)]_8_-O2Oc-NH_2_

FITC-streptavidin (Thermo Fisher Scientific, Waltham, Massachusetts, USA) was incubated with increasing concentrations of polymers with N-terminal biotin (compound 1 or 2) or free amino groups (compound 1a or 2a) at molar ratios of 1:1, 1:2, and 1:4 for one hour at RT. Each complex was separated on a native polyacrylamide gel at a concentration of 0.1 or 0.2 µg per lane with polymer and FITC-Strp as controls. A 15-well Native PAGE 4–16%, Bis–Tris, 1.0 mm, Mini Protein Gel (Thermo Fisher Scientific, Waltham, Massachusetts, USA) was used. The separation conditions were as follows: 50 mM Bis–Tris, 50 mM tricine, pH 6.8 (running buffer), and constant voltage of 150 V for 2 h at 24 °C. Loading buffer composition: 200 mM Bis–Tris, 6 N HCl, 200 mM NaCl, 40% w/v glycerol, 0.004% bromophenol blue, pH 7.2. Cathode buffer composition: 0.4% Coomassie G-250 diluted 10 times in double distilled water in running buffer. At the final step, the gel was placed in 100 mL of fix solution (40% methanol and 10% acetic acid in water) and microwaved at 1100 watts for 45 s. Next, the gel was shaken for 30 min at room temperature. The fixing solution was removed, and the whole procedure was repeated. After this, the gel was ready for visualization.

#### Strp-β-gal complex with Bt-peptidomimetic

The hybrid protein construct streptavidin-β-gal (Thermo Fisher Scientific, Waltham, Massachusetts, USA) at a concentration of 2 mg/mL in H_2_O was incubated for 1 h at RT in molar ratios of 1:1, 1:2, or 1:4 with compound 1 or 2; nonbiotinylated compounds (1a or 2a) were used as controls. All samples were subjected to electrophoretic separation at a protein concentration equal to 7.5 µg per well.

#### Acidic native gel

The polyacrylamide gel was cast and run as follows: stacking gel, 4% acrylamide, pH 5.0 (0.22 M acetic acid/KOH buffer); resolving gel, 8% acrylamide, pH 4.0 (0.075 M acetic acid/KOH buffer); running buffer, 3.55% β-alanine/acetic acid in water, pH 4.0; loading buffer, 50% glycerol in 0.22 M acetic acid/KOH buffer, pH 5.0 with 0.0005% crystal violet.

The gel was run with reverse polarization at 5 mA initially (45 min); then, after the samples migrated out from the stacking gel, it was run at 20 mA (2 h). Images of the gel were taken using a Fusion Fx (Vilber Lourmat, France) system.

### Cell studies

#### FITC-streptavidin: Bt-peptidomimetic complex cellular uptake

Labeled FITC-streptavidin (Thermo Fisher Scientific, Waltham, Massachusetts, USA) at a concentration of 0.2 mg/mL in H_2_O was incubated with peptidomimetic (biotinylated (1 or 2) or lacking biotin (1a or 2a)) at molar ratios of 1:2 and 1:4 for one hour at RT.

All cells were seeded in 24-well plates (initial number of cells: 10^4^) and cultured in 0.5 mL of appropriate medium for 24 h. Next, the cells were washed three times with PBS, and 0.5 mL of peptidomimetic (1, 2 or 1a, 2a) in medium was added. The concentration of the all peptidomimetic (1, 2 or 1a, 2a) was 10 µM (molar ratio 1:2) or 20 µM (molar ratio 1:4) respectively. After the indicated time period (2 h, 4 h or 24 h), the cells were washed with PBS and cultured in FluoroBrite DMEM (Gibco, New York, USA).

An ECLIPSE Ti-E inverted fluorescence microscope (Nikon, Tokyo, Japan) was used to evaluate the complex distribution in the cells. The appropriate filters were used for the detection of DAPI and FITC.

#### Streptavidin-β-gal: Bt-peptidomimetic complex cellular uptake

Streptavidin-β-gal (Invitrogen by Thermo Fisher Scientific, Waltham, Massachusetts, USA) at a concentration of 0.2 mg/mL in H_2_O was incubated with peptidomimetic (biotinylated (1,2) or lacking biotin (1a, 2a)) at molar ratios of 1:2 and 1:4 for 1 h at RT.

**Figure 2:**
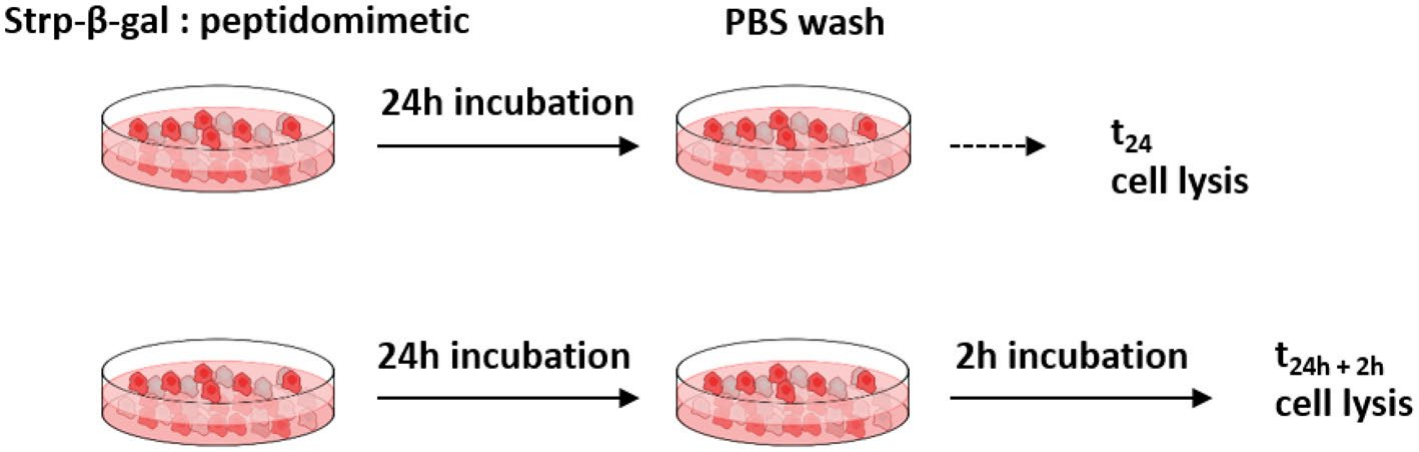
Incubation scheme for Strp-β-gal:peptidomimetics. Additional two hours of culturing allow the membrane bound complex to dissociate.

All cells were seeded in 96-well plates (initial number of cells: 10^3^) and cultured in 0.1 mL of appropriate medium for 48 h. Next, the cells were washed with PBS (three times), and 50 µL of appropriate peptidomimetic (1 or 2) in medium was added. The concentration of each peptidomimetic was 10 µM (molar ratio 1:2) or 20 µM (molar ratio 1:4). Cells were cultured for 24 h and then subjected to PBS washes (2 times). After the selected time period, 0 h (t_24h_) or 2 h (t_24h+2h_) (see scheme on Fig. 2) the cells were washed with PBS and lysed with Reporter Lysis Buffer RLB (Promega, Madison, Wisconsin, USA) according to the manufacturer’s manual. All lysates were immediately snap frozen at − 80 °C.

### Activity studies

The enzymatic activity of β-gal was monitored using resorufin β-d-galactopyranoside (Thermo Fisher Scientific, Waltham, Massachusetts, USA) (final concentration = 2.5 mg/mL dimethyl sulfoxide (DMSO)). The increase in fluorescence was monitored in triplicate using a FluoroStar OMEGA microplate reader (BMG LABTECH, Ortenberg, Germany) with extinction and emission wavelengths of 541 nm and 585 nm. Assay conditions were as follows: 2.5 µL of lysate, 100 µL assay buffer (0.1 M phosphate buffer, pH 7.0) and 5 µL of substrate. The assay was run for 120 min at 37 °C.

### In-cell β-gal activity detection

Cells were incubated with the formed complexes for 24 h in culture medium washed by PBS and cultured for additional 2 h or directly subjected to the next step. After this, two washes with PBS were applied to each system; then, the cells were fixed in 3.7% paraformaldehyde in PBS for 15 min. Next, the cells were washed twice in PBS, and 125 µL of reaction mixture was added to each system. The reaction buffer consisted of 40 mM citric acid, 40 mM sodium phosphate, 300 mM NaCl, 10 mM mercaptoethanol, and 4 mM MgCl_2_ (pH 6.0) with 2 mM X-Gal (5-bromo-4-chloro-3-indolyl β-d-galactopyranoside) added immediately prior to use from a 34 mM stock in DMSO^17^. The plate was incubated for two hours at 37 °C and observed under an Olympus IX51 microscope (Olympus, Tokyo, Japan). Application of the X-Gal reagent in the presence of β-gal activity results in a blue, insoluble product that precipitates and can be monitored under an optical microscope.

### Cytotoxicity studies

All cell lines were seeded at a concentration of 5 × 10^3^ per well in 96-well plates and cultured for 48 h in 100 µL of medium. Next, the medium was replaced with new medium containing the target amount of tested compounds or their complexes.

The following compounds were tested: Bt-O2Oc-[Dap(GO2)]_6_-O2Oc-NH_2_ (Compound **1**), where Bt—biotin, O2Oc—8-amino-3,6-dioxaoctanoic acid, Dap—L-2,3-diaminopropionic acid, GO2—8-amidino-3,6-dioxaoctanoic acid, Bt-O2Oc-[Dap(GO2)]_8_-O2Oc-NH_2_ (Compound **2**), O2Oc-[Dap(GO2)]_6_-O2Oc-NH_2_ (Compound **1a**) and O2Oc-[Dap(GO2)]_8_-O2Oc-NH_2_ (Compound **2a**) at concentrations of 1, 5, 10, 20, 50, and 100 µM in the well;

- Complex FITC-Strp: Compound **1**/Compound **2** and FITC-Strp: Compound **1a**/Compound **2a** in molar ratios of 1:2 and 1:4 at concentrations of 1, 10, 20, and 30 µM in the well;
- Complex Strp-β-gal: Compound **1**/Compound **2** and complex Strp-β-gal: Compound **1a**/Compound **2a** in molar ratios of 1:2 and 1:4 at concentrations of 1, 10, and 20 µM in the well.

Each incubation was run in triplicate for 24 h. Untreated cells were used as a control. After 24 h, the medium was removed, and the cells were incubated with MTT-containing medium for 4 h. Next, DMSO was added to each well, and the results were read using a plate reader (SPECTROstar Nano, BMG LABTECH, Ortenberg, Germany) at two different wavelengths, 570 nm and 690 nm.

### Immunofluorescence staining and analyses of β-gal in selected cell lines

HB2 or MDA-MB-231 cells were seeded and grown on glass slides (removable 3-well chamber, IBIDI, Gräfelfing, Germany). On the day of the experiment, cells were incubated with a freshly prepared complex of Strp-β-gal and the chosen compound. At the appropriate time points (t_24h_ and t_24h+2h_)_,_ the cells were washed and subjected to further experimental procedures.

After incubation, the cells were fixed for 10 min in 1% paraformaldehyde in PBS, washed in PBS, permeabilized in 1% Triton X-100 in PBS, and blocked for 4 h with blocking buffer consisting of 2% FBS, 2% bovine serum albumin (BSA) and 0.2% fish gelatin in PBS (lacking Ca^2+^ and Mg^2+^). Next, the cells were incubated for 1 h with β-galactosidase monoclonal primary antibodies (Thermo Fisher Scientific, Waltham, Massachusetts, USA, cat number MA1-152; 1:50), washed four times in 0.1% Tween in PBS followed by DyLight^TM^488-conjugated goat anti-mouse IgG secondary antibody diluted in blocking buffer for 45 min (Thermo Fisher Scientific, Waltham, Massachusetts, USA, cat number 35502; 1:50). After 30 min of incubation, Hoechst reagent was added to all systems. After 45 min of incubation, the cells were washed four times in 0.1% Tween in PBS. Blocking solution was used instead of primary antibody as a control for nonspecific staining. Coverslips were mounted on the slides using Fluoromount Aqueous Mounting Medium (Sigma‒Aldrich, Darmstadt, Germany). The specimens were imaged with an ECLIPSE Ti-E confocal laser scanning microscope. To determine the mean fluorescence intensity profile of the immunocomplexes, the mean intensity values for green channels was analyzed using NIS-Elements software (Nikon, Tokyo, Japan) with the Automated Measurement Results option.

### Statistical analyses

The statistical analyses were performed using GraphPad Prism 8 (Graphpad software, USA). The Shapiro–Wilk test was used to determine normality of the datasets. For data with a normal distribution, the unpaired *t*-test was used. For other cases, the nonparametric Mann–Whitney test was applied. The results are expressed as mean ± SEM. Values of *p* ≤ 0.05 were considered statistically significant.

## Results

To determine if the biotinylated compounds are able to form complex with protein conjugated streptavidin, we first aimed to confirm the presence of the complex formed by means of polyacrylamide electrophoresis, followed by cytotoxicity study of compounds and formed complexes. The final experiments were designed to provide the information regarding efficacy of transport across the cell membrane of model enzyme.

### Synthesis

Two DAPEG polymers consisting of six (Compound 1) or eight (Compound 2) monomers were synthesized and labeled with an N-terminal biotin. Synthesis were performed on solid support using combined synthesis system. Initially DAPEG polymers with 6 or 8 mer length were synthesized using microwave irradiation. After this stage the side chain protecting group were removed and appropriate oxa acid with guanidine group were attached to free amino group of polymer. The molecular weights of the compounds were nearly identical to the calculated weights and ranged from 1947.1 to 2719.3 Da (Table 1). It is generally known that biotin-containing compounds display exceptionally high affinity toward streptavidin or avidin, reaching a subpicomolar dissociation constant.

**Table 1:** Physiochemical characteristics of peptidomimetic compounds.

No.	Sequence	Retention time* [min]	Molecular weight calculated/determined (M + H^+^)[Da]**	Refs.
1	Bt-O2Oc-[Dap(GO2)]_6_-O2Oc-NH_2_	7.46	2174.0/2173.1	–
2	Bt-O2Oc-[Dap(GO2)]_8_-O2Oc-NH_2_	11.77	2720.0/2719.3	–
1a	O2Oc-[Dap(GO2)]_6_-O2Oc-NH_2_	3.40	1947.1/1947.1	^14^
2a	O2Oc-[Dap(GO2)]_8_-O2Oc-NH_2_	2.97	2493.7/2493.5	^14^

*UPLC analysis (Nexera X2 LC-30AD [Shimadzu, Kyoto, Japan] equipped with a Phenomenex column (150 × 2.1 mm) with grain size 1.7 µm (peptide XB-C18) equipped with a UV‒Vis detector and a fluorescence detector). Flow rate 0.3 mL/min. A linear gradient from 2 to 80% B within 15 min was applied (A: 0.1% trifluoroacetic acid; B: 80% acetonitrile in A).

**HR MALDI analysis with 2,5-dihydroxybenzoic acid as a matrix.

### Complex formation

**Figure 3:**
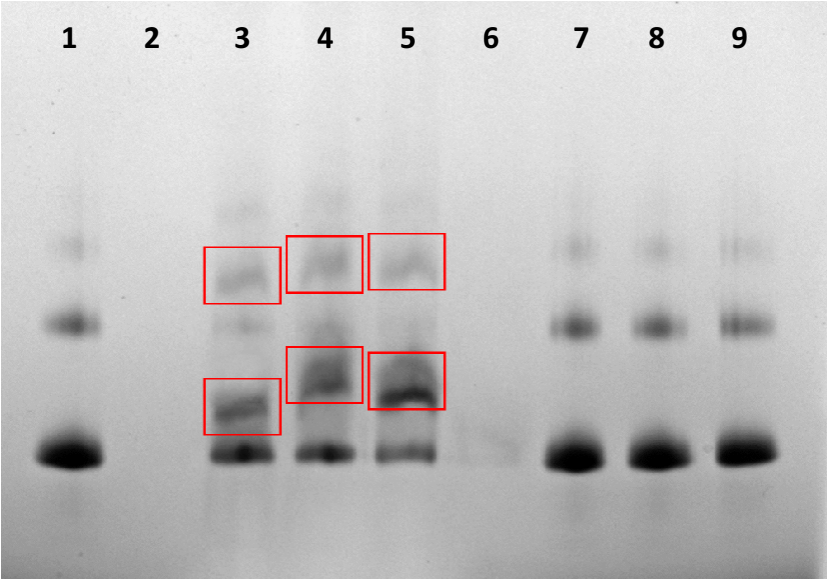
Native gel electrophoresis of a complex. Lane **1**: FITC-Strp, lane **2**: Compound 1; lane **3**: complexed FITC-Strp:Compound 1 in a molar ratio of 1:1; lane **4**: complexed FITC-Strp:Compound 1 in a molar ratio of 1:2; lane **5**: complexed FITC-Strp:Compound 1 in a molar ratio of 1:4. Lane **6**: Compound 1a; lane **7**: complexed FITC-Strp:Compound 1a in a molar ratio of 1:1; lane **8**: complexed FITC-Strp:Compound 1a in a molar ratio of 1:2; lane **9**: complexed FITC-Strp:Compound 1a in a molar ratio of 1:4.

**Figure 4:**
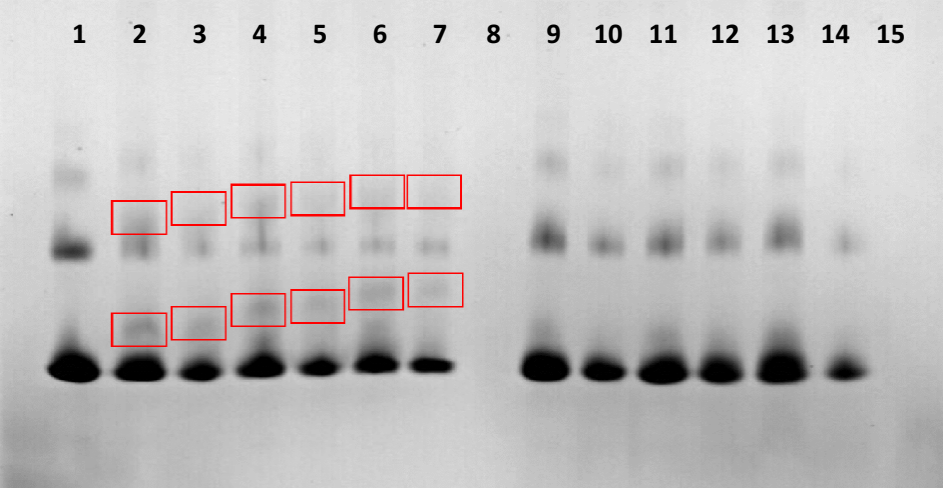
Native gel electrophoresis of a complex. Lane **1**: FITC-Strp (0.2 µg), lane **2**: complexed FITC-Strp:Compound 2 in a molar ratio of 1:1 (0.2 µg); lane **3**: complexed FITC-Strp:Compound 2 in a molar ratio of 1:1 (0.1 µg); lane **4**: complexed FITC-Strp:Compound 2 in a molar ratio of 1:2 (0.2 µg); lane **5**: complexed FITC-Strp:Compound 2 in a molar ratio of 1:2 (0.1 µg); lane **6**: complexed FITC-Strp:Compound 2 in a molar ratio of 1:4 (0.1 µg); lane **7:** complexed FITC-Strp:Compound 2 in a molar ratio of 1:4 (0.2 µg); lane **8**: Compound 2. Lane **9**: complexed FITC-Strp:Compound 2a in a molar ratio of 1:1 (0.2 µg); lane **10**: complexed FITC-Strp:Compound 2a in a molar ratio of 1:1 (0.1 µg); lane **11**: complexed FITC-Strp:Compound 2a in a molar ratio of 1:2 (0.2 µg); lane **12**: complexed FITC-Strp:Compound 2a in a molar ratio of 1:2 (0.1 µg); lane **13**: complexed FITC-Strp:Compound 2a in a molar ratio of 1:4 (0.2 µg); lane **14**: complexed FITC-Strp:Compound 2a in a molar ratio of 1:4 (0.1 µg); lane **15**: Compound 2a.

Next, we aimed to verify the ability of the abovementioned compounds to form a complex with fluorescently labeled streptavidin (FITC-Strp). Electrophoretic separation of a mixture composed of Compound 1 or 1a and 2 or 2a with FITC-Strp at three different ratios (1:1, 1:2 or 1:4) resulted in the appearance of additional multiple bands (Fig. 3, boxes in lanes 3–5). In Fig. 4, the same extra bands were observed (boxes in lanes 2–7).

### Cytotoxicity

**Figure 5:**
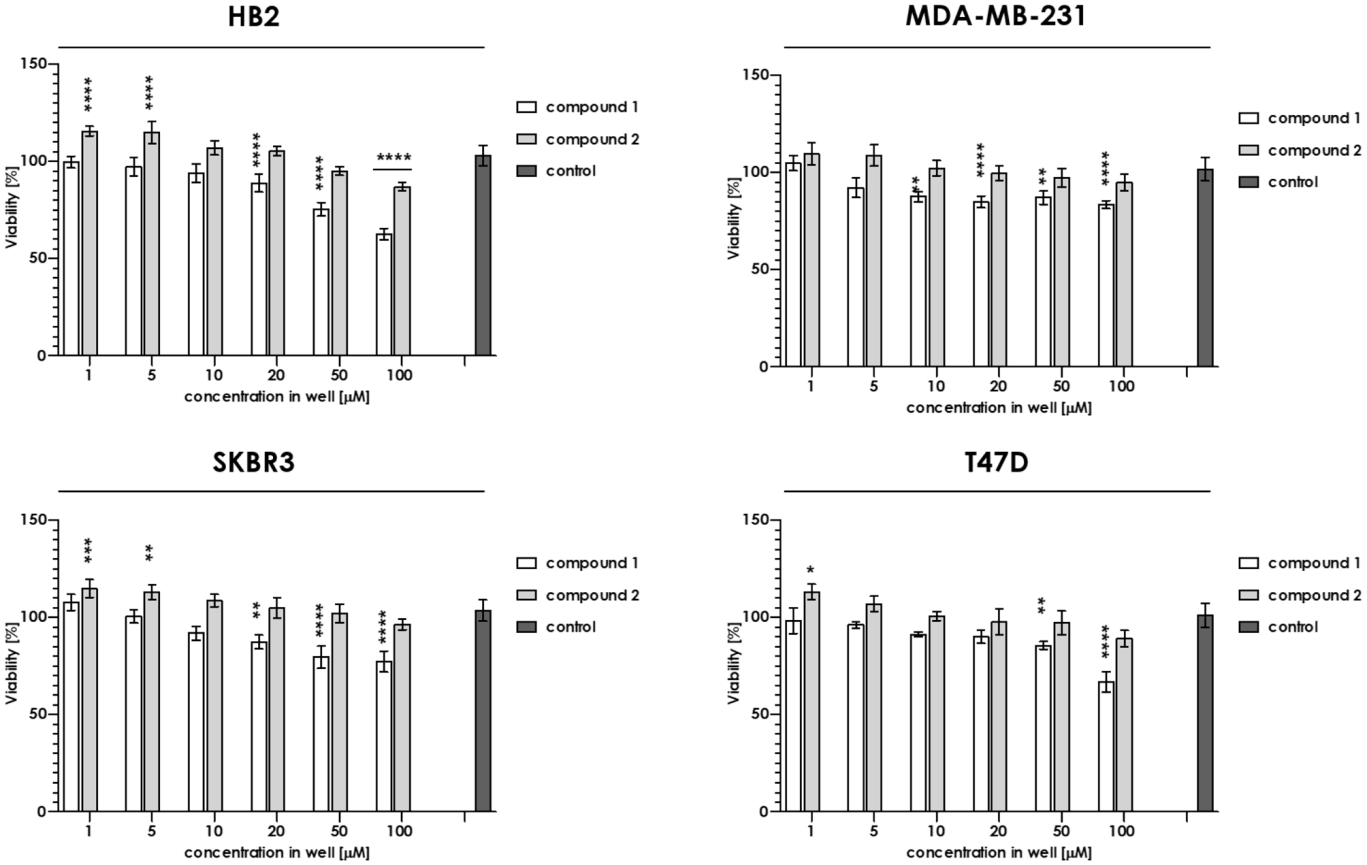
Cytotoxicity of Compounds 1 and 2 against a panel of cell lines: HB2, MDA-MB-231, SKBR3 and T47D (mean ± SD). Statistical comparisons were performed using one-way ANOVA: *p < 0.05; **p < 0.01; ***p < 0.001; ****p < 0.0001 (number of samples in one system, n = 6).

**Figure 6:**
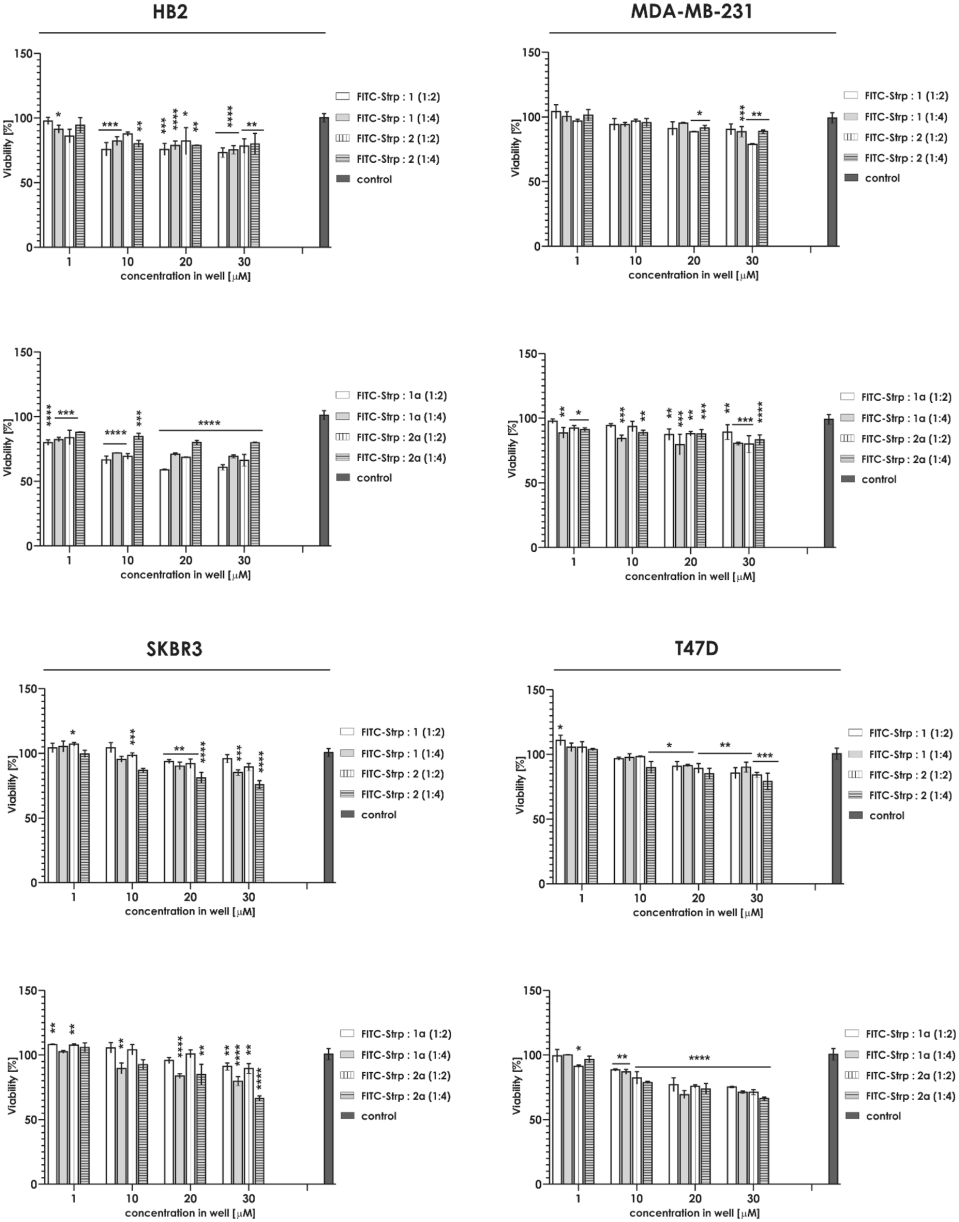
Cytotoxicity of Compounds 1 and 2 mixed with FITC-Strp against a panel of cell lines: HB2, MDA-MB-231, SKBR3 and T47D (mean ± SD). Statistical analysis was performed using one-way ANOVA: *p < 0.05; **p < 0.01; ***p < 0.001; ****p < 0.0001 (number of samples in one system, n = 6).

Our results suggest that the complexes were successfully formed. Next we proceed to determine whether such complexes are able to translocate into the selected cell lines without significant cytotoxicity. Analysis of the cytotoxicity profiles of Compound 1 against a panel of breast cancer cell lines (MDA-MB-231, T47D and SKBR3) and the noncancerous control (HB2) indicated that neither the formed complex nor its constituents significantly impacted cell viability at concentrations up to 20 µM. See Figs. 5 and 6.

**Figure 7:**
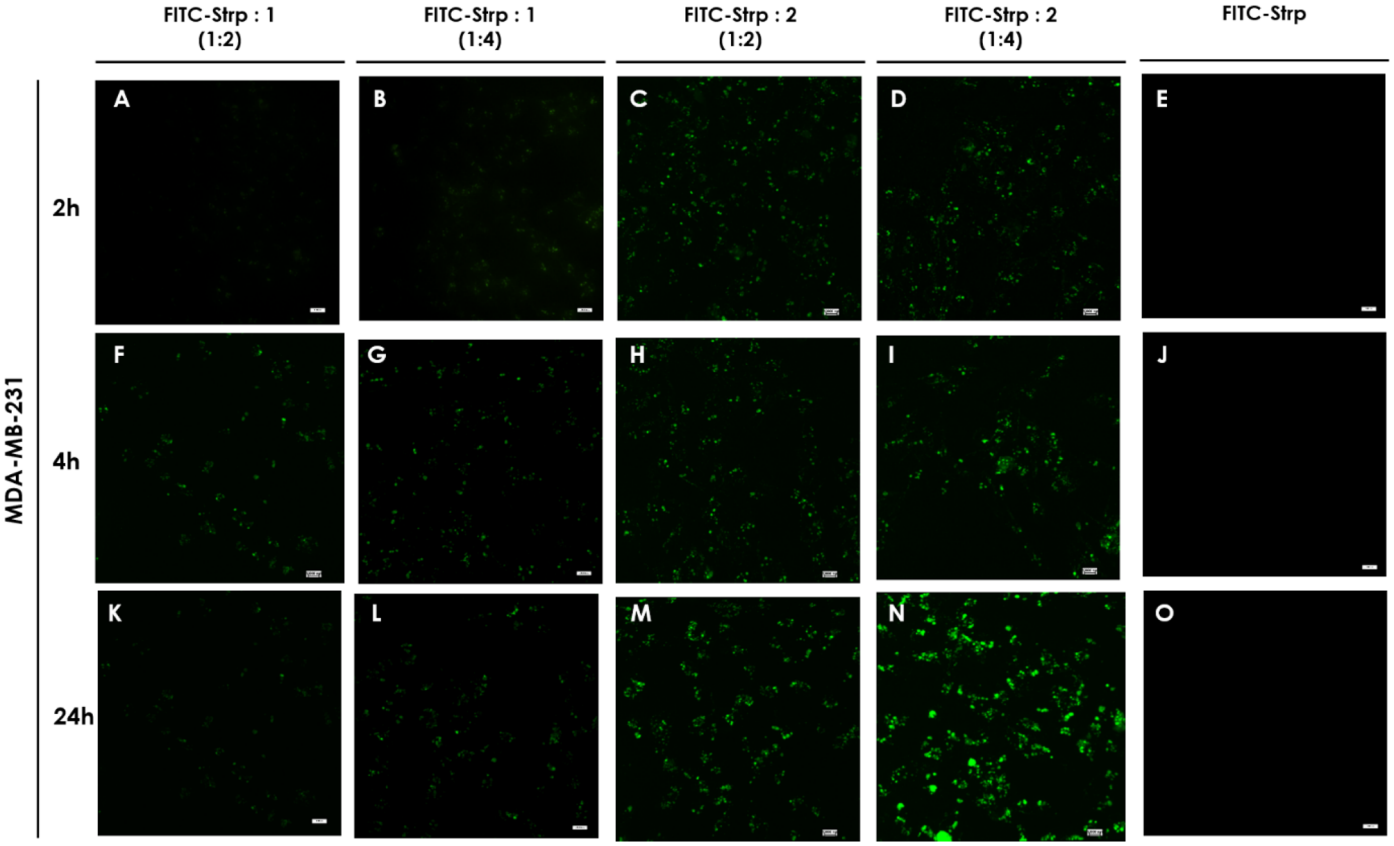
Confocal fluorescence microscopy images of MDA-MB-231 cells incubated with complexes. (**A**, **E**, **I**)—FITC-Strp:Compound 1 in a molar ratio of 1:2; (**B**, **F**, **J**)—FITC-Strp:Compound 1 in a molar ratio of 1:4; (**C**, **G**, **K**)—FITC-Strp:Compound 2 in a molar ratio of 1:2; (**D**, **H**, **L**)—FITC-Strp:Compound 2 in a molar ratio of 1:4. (**A–D**)—incubation time 2 h, (**E–H**)—incubation time 4 h, (**I–L**)—incubation time 24 h. Magnification 20 × scale bar 10 µm.

**Figure 8:**
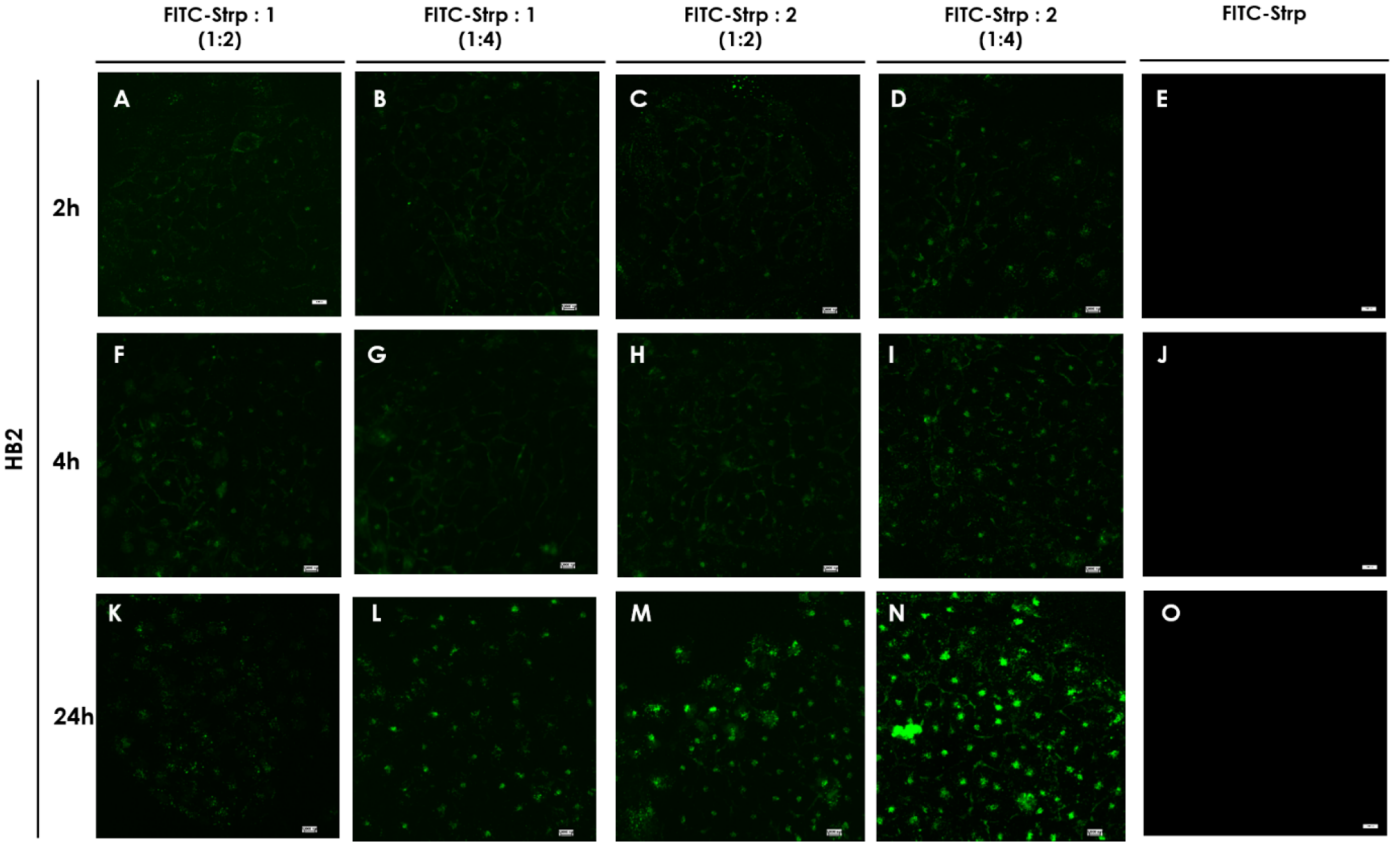
Confocal fluorescence microscopy images of HB2 cells incubated with complexes. (**A**, **E**, **I**)—FITC-Strp:Compound 1 in a molar ratio of 1:2; (**B**, **F**, **J**)—FITC-Strp:Compound 1 in a molar ratio of 1:4; (**C**, **G**, **K**)—FITC-Strp:Compound 2 in a molar ratio of 1:2; (**D**, **H**, **L**)—FITC-Strp:Compound 2 in a molar ratio of 1:4. (**A–D**)—incubation time 2 h, (**E–H**)—incubation time 4 h, (**I–L**)—incubation time 24 h. Magnification 20 × scale bar10 µm.

**Figure 9:**
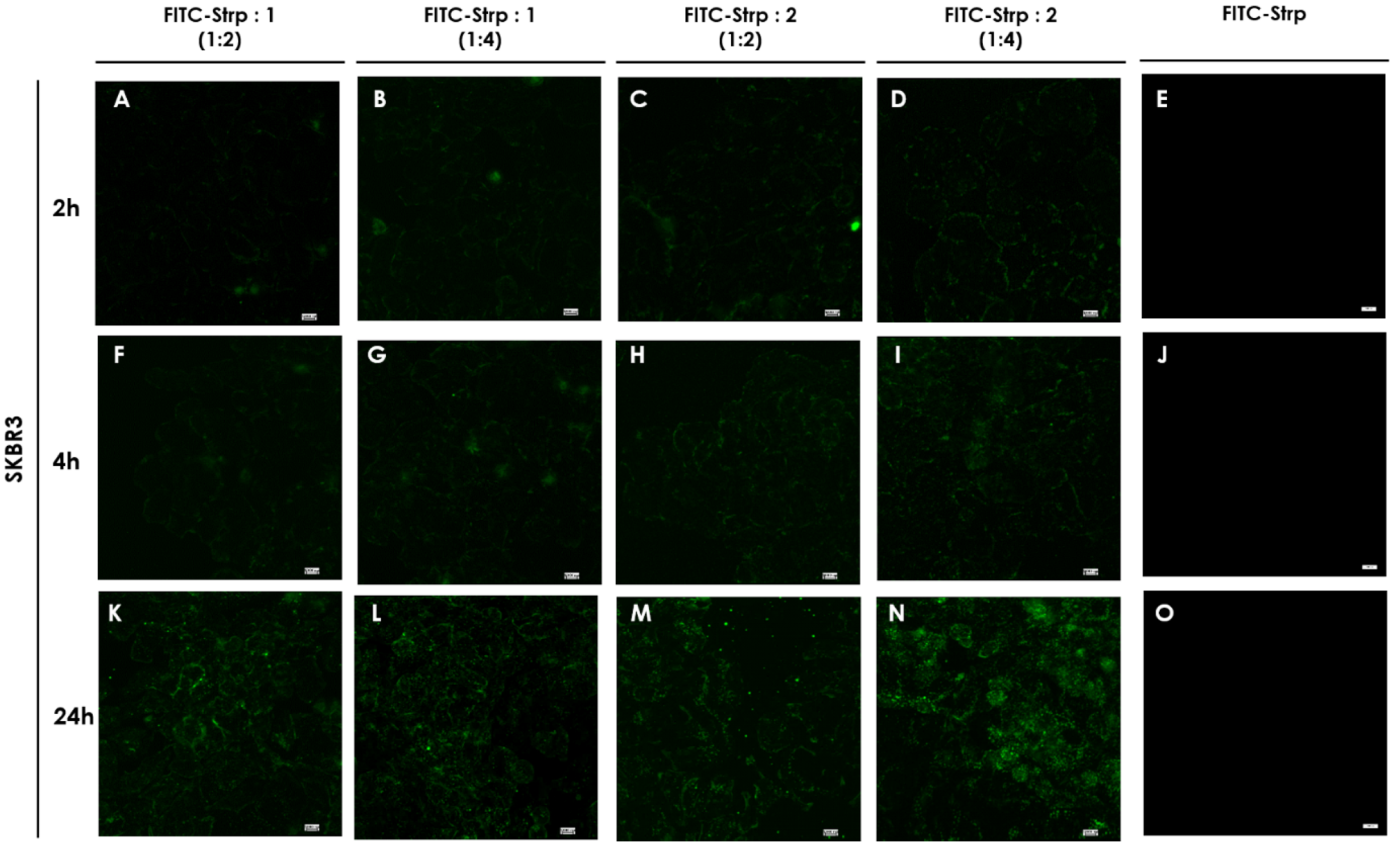
Confocal fluorescence microscopy images of SKBR3 cell lines incubated with complexes. (**A**, **E**, **I**)—FITC-Strp:Compound 1 in a molar ratio of 1:2; (**B**, **F**, **J**)—FITC-Strp:Compound 1 in a molar ratio of 1:4; (**C**, **G**, **K**)—FITC-Strp:Compound 2 in a molar ratio of 1:2; (**D**, **H**, **L**)—FITC-Strp:Compound 2 in a molar ratio of 1:4. **A–D**—incubation time 2 h, (**E–H**)—incubation time 4 h, (**I–L**)—incubation time 24 h. Magnification 20× scale bar 10 µm.

**Figure 10:**
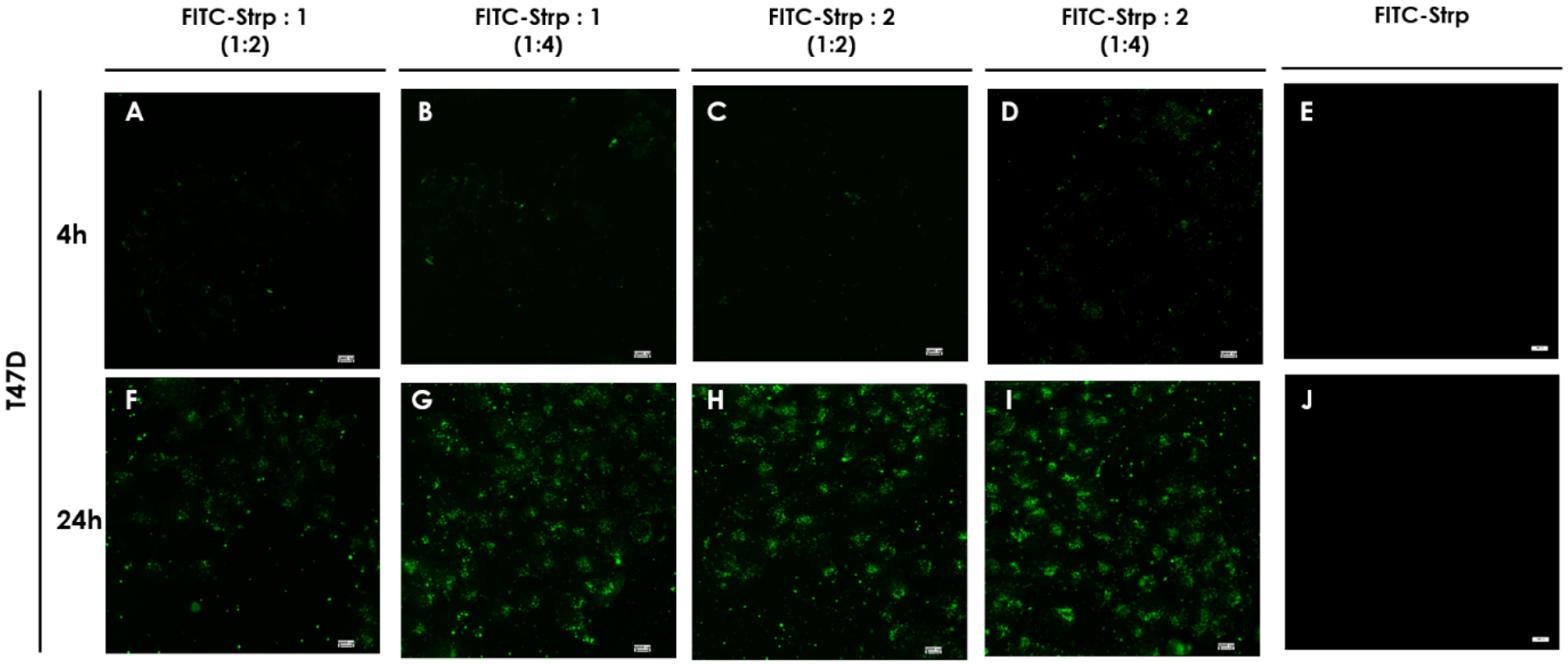
Confocal fluorescence microscopy images of T47D cell lines incubated with complexes. (**A**, **E**)—FITC-Strp:Compound 1 in a molar ratio of 1:2; (**B**, **F**)—FITC-Strp:Compound 1 in a molar ratio of 1:4; (**C**, **G**)—FITC-Strp:Compound 2 in a molar ratio of 1:2; (**D**, **H**)—FITC-Strp:Compound 2 in a molar ratio of 1:4. (**A–D**)—incubation time 4 h, (**E–H**)—incubation time 24 h. Magnification 20 × scale bar 10 µm.

Based on the above results, we incubated the abovementioned cell lines with FITC-Strp + Compound **1** or **2** in stoichiometric ratios of 1:2 or 1:4 at concentrations (referred to as protein amounts) equal to 10 µM (molar ratio 1:2) or 20 µM (molar ratio 1:4). As shown in Figs. 7, 8, 9 and 10, green fluorescence indicating the presence of FITC-Strp was observed in all cell lines.

No fluorescence was observed for any control system, including FITC alone and FITC-Strp alone. Localization of the signal differed among cell lines and incubation time For HB2, membrane and nuclear localization dominate, whereas for the cancerous cell lines, fluorescence is visible in the cytoplasmic/membrane compartment (Fig. 8). The fluorescence exhibited the highest level of intensity in all systems following a 24-h incubation period, while the efficacy appeared to be insufficient after 2 and 4 h. This observation is pivotal for the purpose of strategizing subsequent experiments, and as a result, we have opted to utilize a 24-h incubation period. The previous reports on CPP utilization indicated that the incubation periods ranged from 1 to 4 h were effective^18,19^. However, it is important to note that this time is highly influenced by the specific cell types employed and other nuanced factors.

The in-cell fluorescence measured for all tested systems indicates that Bt-O2Oc-[Dap(GO2)]_8_-O2Oc-NH_2_ is a better mediator of labeled protein translocation than Bt-O2Oc-[Dap(GO2)]_6_-O2Oc-NH_2_ (7, 8, 9 and 10). The obtained results correspond well with several reports describing the efficient implementation of several biotinylated CPPs complexed with fluorescently labeled streptavidin or avidin. Numerous CPPs have been used with different combinations of fluorescent labels^20,21^.

Next, we analyzed whether we were able to translocate model proteins with enzymatic activity using these systems, with streptavidin β-galactosidase (Strp-β-gal) conjugate as the selected model. β-Gal is a protein formed by four subunits, each with 1023 amino acid residues, and is frequently utilized as a reporter molecule. The enzyme hydrolyzes lactose and other β-D-galactosides into monosaccharides. Although very specific for the galactose moiety, it tolerates a variety of structures elsewhere in the substrate; thus, any number of organic dyes with a galactose moiety can be used as reporters. For the activity study, we utilized resorufin β-D-galactopyranoside, which produces free resorufin upon hydrolysis. For the cellular study, X-Gal was used.

**Figure 11:**
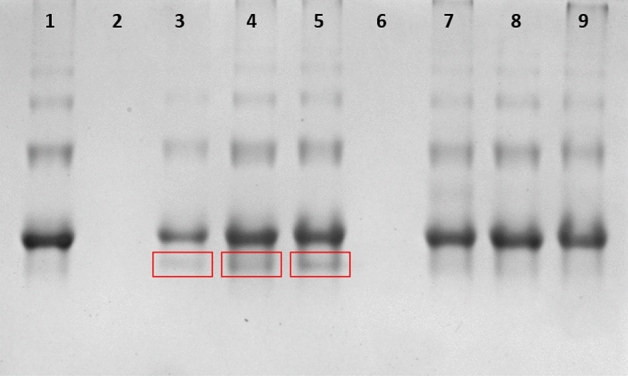
Native gel electrophoresis of Strp-β-gal:Compound 1 (lanes **3**–**5**) and Strp-β-gal:Compound 1a (lanes **7**–**9**) in acidic conditions. Lane **1**: Strp-β-gal; lane **2**: Compound 1; lane **3**: complex Strp-β-gal:Compound 1 in a molar ratio of 1:1; lane **4**: complex Strp-β-gal:Compound 1 in a molar ratio of 1:2; lane **5**: complex Strp-β-gal:Compound 1 in a molar ratio of 1:4; lane **6**: Compound 1a; lane **7**: mixture Strp-β-gal:Compound 1a in a molar ratio of 1:1; lane **8**: mixture Strp-β-gal:Compound 1a in a molar ratio of 1:2; lane **9**: mixture Strp-β-gal:Compound 1a in a molar ratio of 1:4.

**Figure 12:**
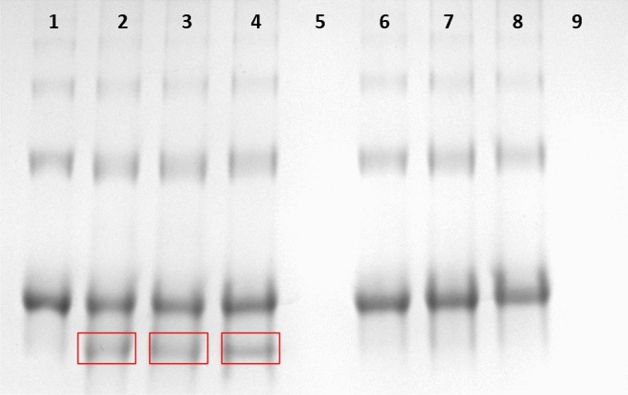
Native gel electrophoresis in acidic conditions of the Strp-β-gal complex: Compound 2 (lanes **2**–**4**) and Strp-β-gal mixture: Compound 2a (lanes **6**–**8**). Lane **1**: Strp-β-gal; lane **2**: complex Strp-β-gal: Compound 2 in a molar ratio of 1:1; lane **3**: complex Strp-β-gal: Compound 2 in a molar ratio of 1:2; lane **4**: complex Strp-β-gal: Compound 2 in a molar ratio of 1:4; lane **5**: Compound 2; lane **6**: mixture Strp-β-gal: Compound 2a in a molar ratio of 1:1; lane **7**: mixture Strp-β-gal: Compound 2a in a molar ratio of 1:2; lane **8**: mixture Strp-β-gal: Compound 2a in a molar ratio of 1:4; lane **9**: Compound 2a.

To verify the efficacy of complex formation, we performed electrophoretic separation in native polyacrylamide gel with reverse polarization under acidic conditions. This analysis indicated the presence of a complex of Strp-β-gal and one of the biotinylated peptidomimetics Bt-O2Oc-[Dap(GO2)]_6_-O2Oc-NH_2_ or Bt-O2Oc-[Dap(GO2)]_8_-O2Oc-NH_2_ (red boxes) (Fig. 11 (lanes 3–5) and Fig. 12 (lanes 2–4), respectively). Above results are in parallel with those obtained for FITC-Strp conjugates. It indicates that various modifications of streptavidin did not altered the ability to biotin. With this proof of mutual and selective interactions of these two molecules, we proceeded to investigate the impact of the formed complexes on selected cell lines.

**Figure 13:**
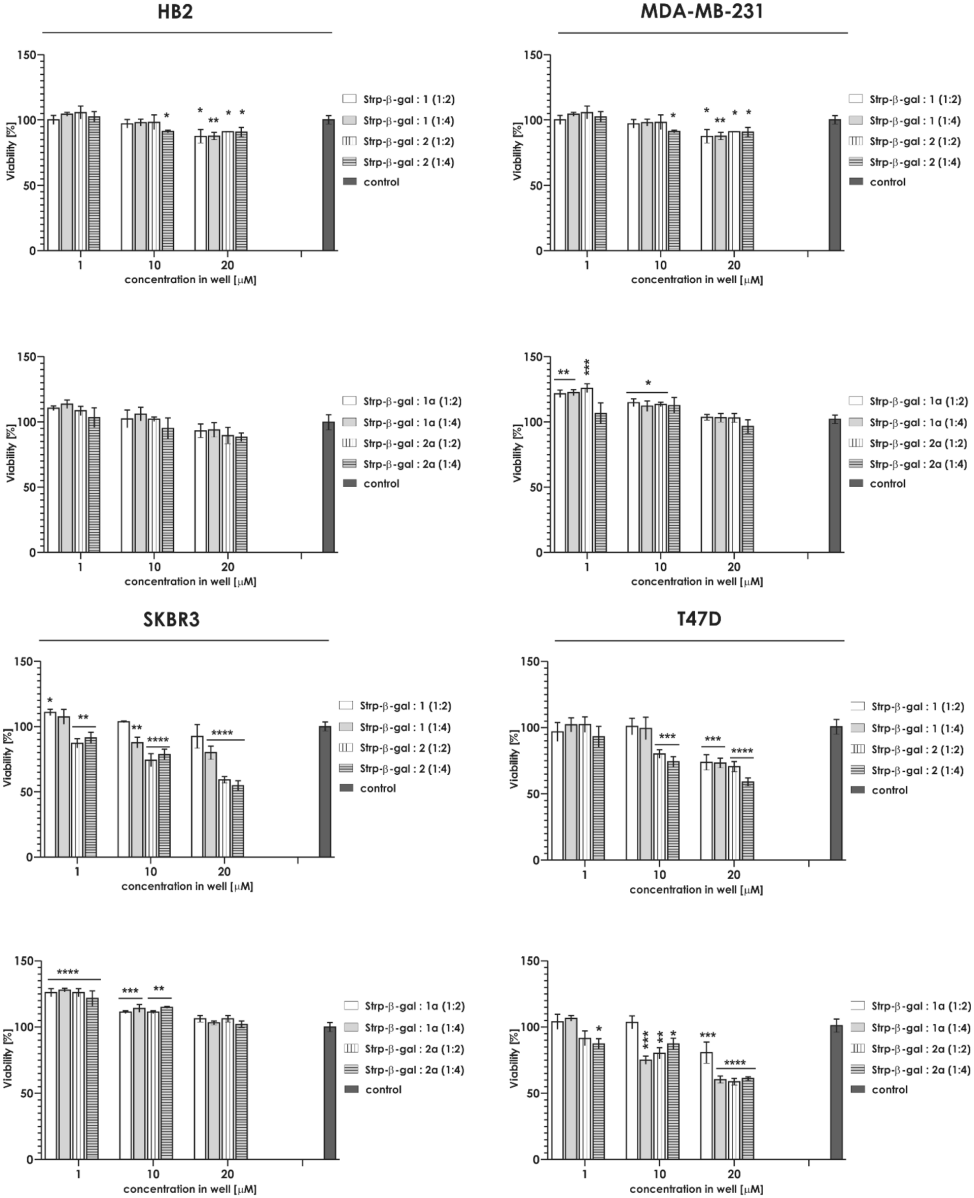
Cytotoxicity of Compounds 1/1a and 2/2a mixed with Strp-β-gal against HB2, MDA-MB-231, SKBR3 and T47D cells (mean ± SD). Statistical calculations were performed using one-way ANOVA: *p < 0.05; **p < 0.01; ***p < 0.001; ****p < 0.0001 (number of samples in one system, n = 6).

The analysis of cytotoxicity against the selected cell lines indicated that HB2 and MDA-MB-231 viability was not significantly influenced by the presence of the tested complexes at concentrations up to 20 µM. Above this concentration, a 20–30% reduction in cell number was observed. For T47D or SKBR3, the complexes significantly reduced the number of live cells at concentrations starting from 10 µM (Fig. 13). These data forced us to exclude T47D and SKBR3 cell lines from further experiments.

Delivery the active enzyme to the cells.

**Figure 14:**
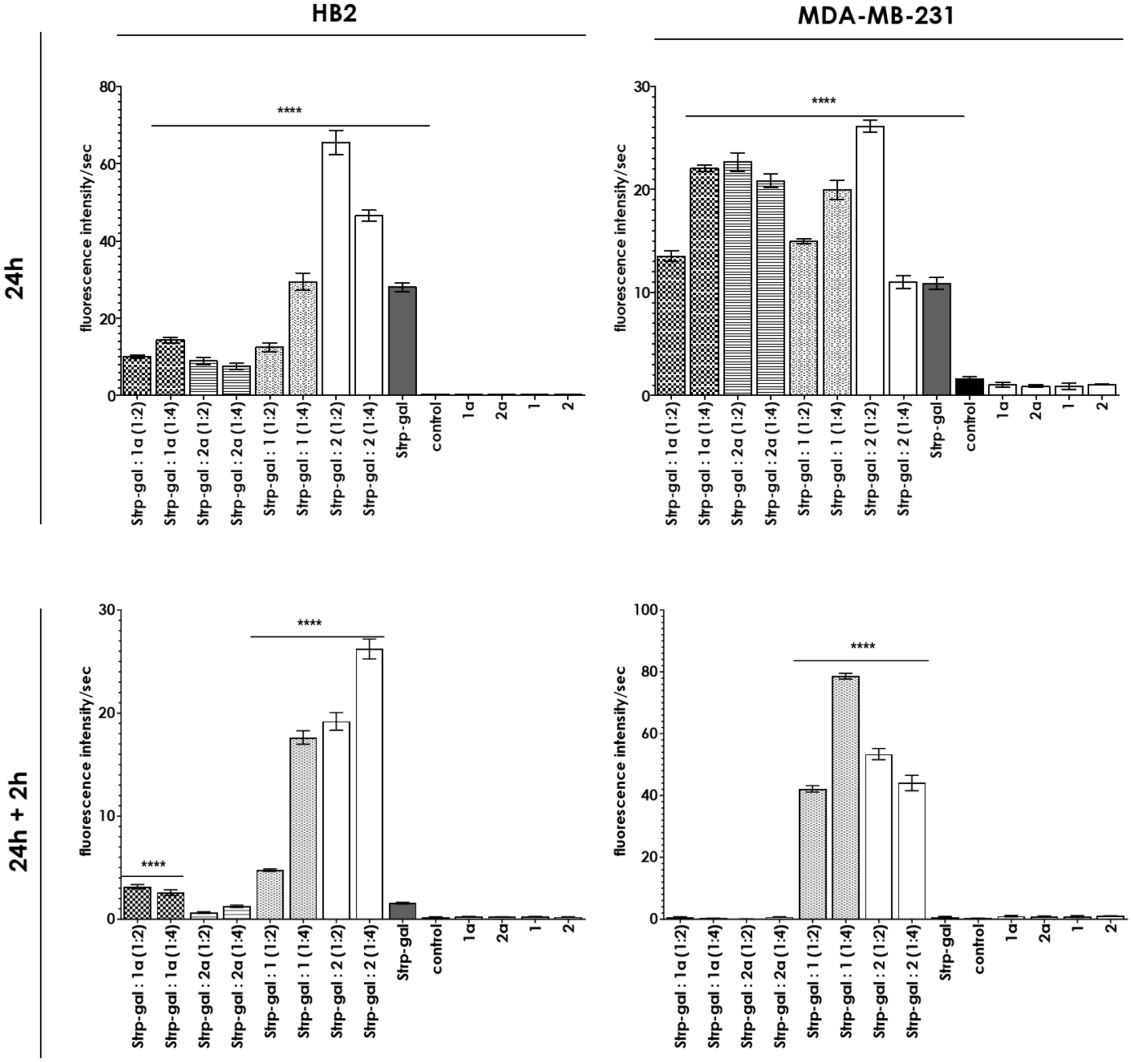
Activity of β-gal in cell lysates of HB2 or MDA-MB-231 cells incubated for 24 h with the indicated compound or mixtures (time point t_24h_) and 24 h followed by 2 h of culture in fresh medium (time point t_24h + 2h_) (mean ± SD; n = 3). Statistical calculations were performed using one-way ANOVA: ****p < 0.0001.

Incubation of the formed Strp-β-gal:Bt-O2Oc-[Dap(GO2)]_6/8_-O2Oc-NH_2_ complexes with cell lines followed by β-galactosidase activity assays using a fluorescent β-gal substrate (resorufin β-D-galactopyranoside) indicated that after 24 h of incubation, β-gal activity was observed in the majority of the systems, with different activity levels (see Fig. 14).

The greatest fluorescence increase was observed for systems including biotinylated polymers, with increases 2–6-fold greater than those for nonbiotinylated compounds. Culturing the cells for an additional 2 h, resulted in a significant reduction in the activity of β-gal in all systems other than those with complexes formed with biotinylated polymers. The level of recorded enzymatic activity was several times greater than that of the control systems. In MDA-MB-231 cell line the unexpected fluorescence increase is recorded. We are not able to provide reasonable explanations of this cell specific issue.

**Figure 15:**
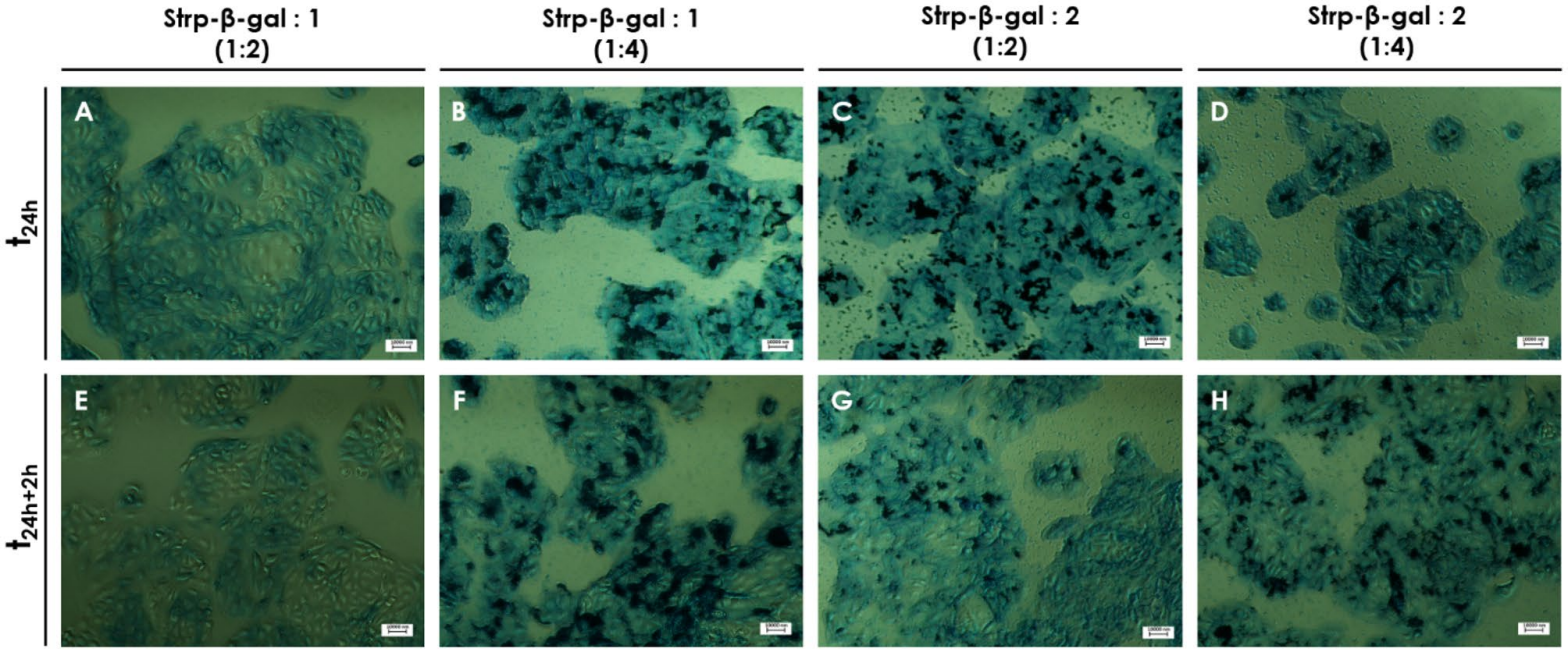
Light microscopy images of HB2 cells after 24 h of incubation with the following mixtures: (**A, E**)—Strp-β-gal:Compound 1 (molar ratio 1:2); (**B, F**)—Strp-β-gal:Compound 1 (molar ratio 1:4); (**C, G**)—Strp-β-gal:Compound 2 (molar ratio 1:2); (**D, H**)—Strp-β-gal:Compound 2 (molar ratio 1:4). (**A–D**)—t_24h_, (**E–H**)—t_24h+2h_. Magnification 20 × scale bar 10 µm.

**Figure 16:**
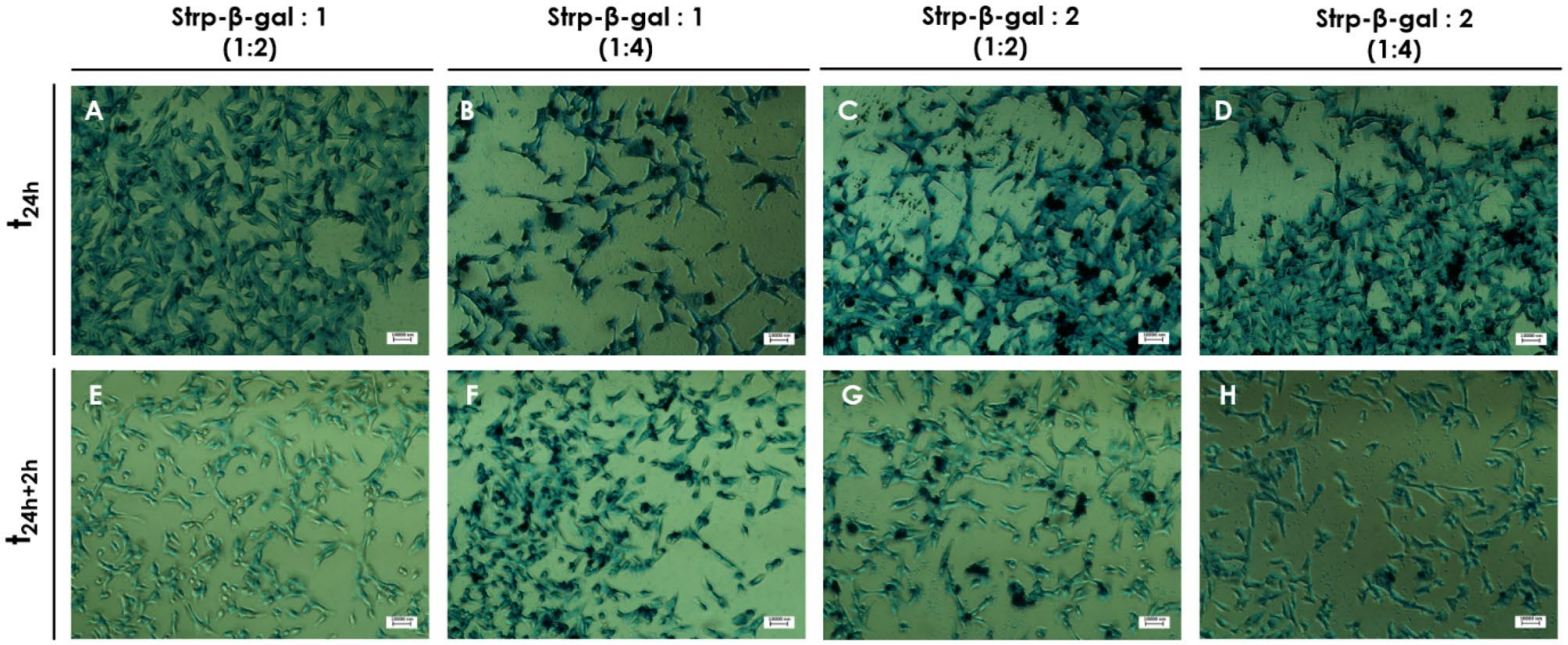
Light microscopy images of MDA-MB-231 cells after 24 h of incubation with the following mixtures: (**A**, **E)**—Strp-β-gal:Compound 1 (molar ratio 1:2); (**B, F)**—Strp-β-gal:Compound 1 (molar ratio 1:4); (**C, G)**—Strp-β-gal:Compound 2 (molar ratio 1:2); (**D, H)**—Strp-β-gal:Compound 2 (molar ratio 1:4). (**A**–**D)**—t_24h_, (**E**–**H)**—t_24h+2 h_. Magnification 20 × scale bar 10 µm.

**Figure 17:**
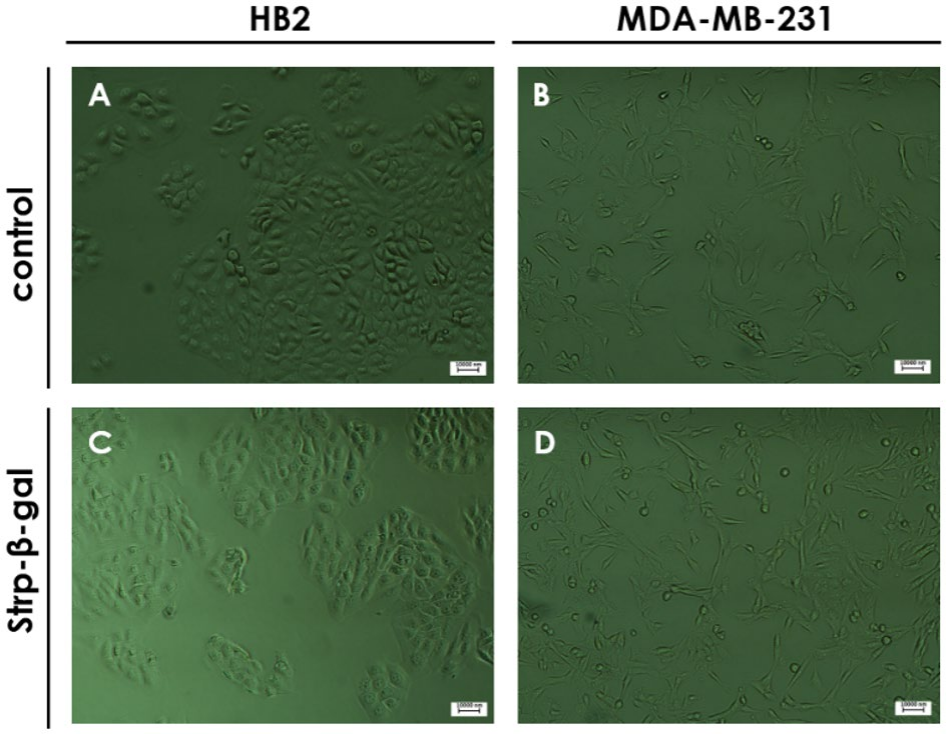
Light microscopy images of HB2 and MDA-MB-231 cells after 24 h of incubation with control (**A**, **B**) or Strp-β-gal (**C**, **D**). Magnification 20 × scale bar 10 µm.

These observations were confirmed by microscopy observations of intracellular β-gal substrate (X-gal) (Figs. 15, 16); that is, blue color resulting from X-gal enzymatic breakdown was visible on membrane compartment and inside the tested cell lines at all time points, confirming the presence of active enzyme’ form. No blue spots were observed for control—nontreated cell lines (Fig. 17). However, some minimal background blue color was observed as the result of endogenous and transported β-gal, since X-gal is a universal substrate for any β-galactosidase activity.

**Figure 18:**
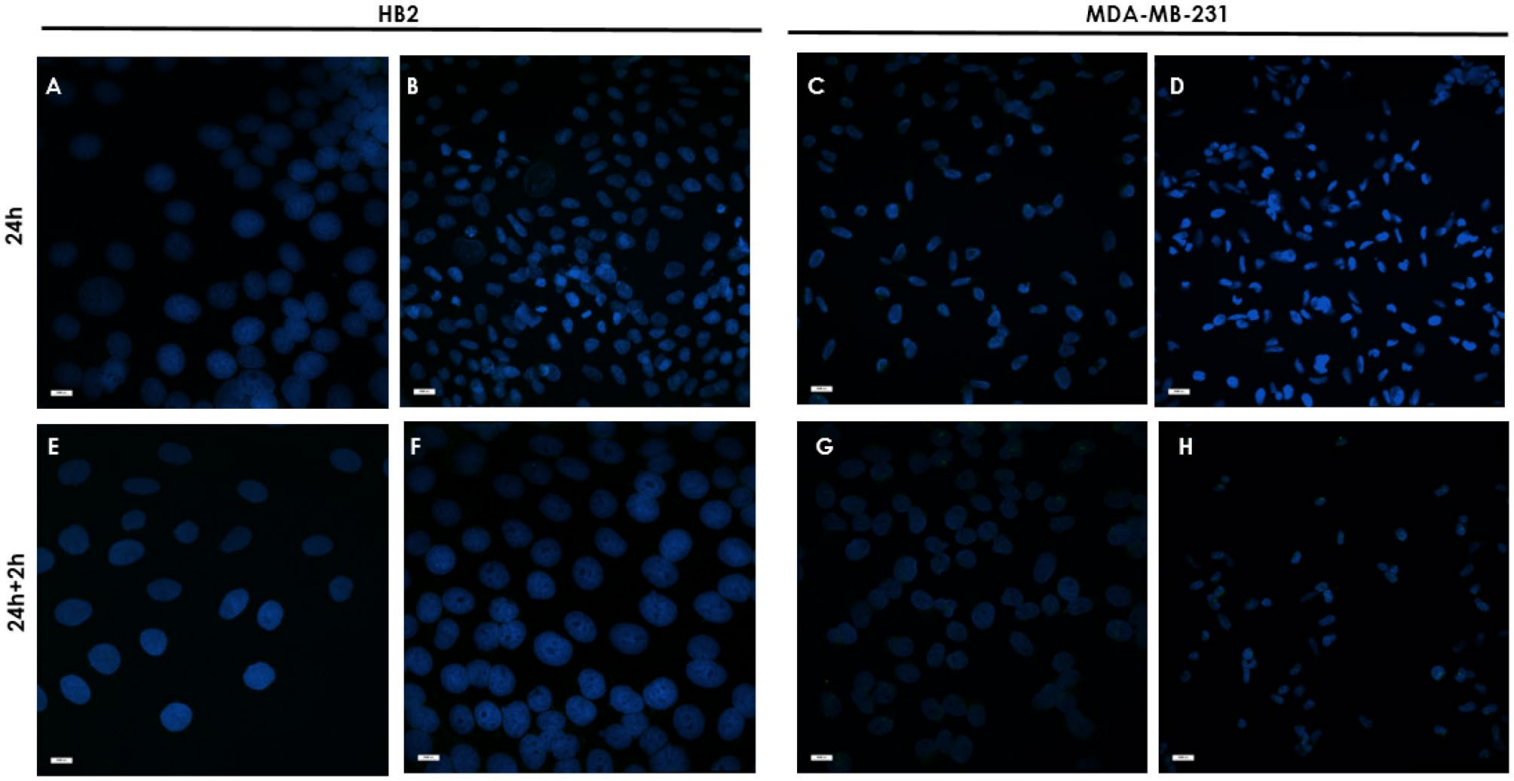
Immunofluorescence staining of HB2 and MDA-MB-231 cell cultures. (**A**, **E**, **C**, **G**)—control; (**B, F, D, H**)—incubated with Strp-β-gal; (**A**–**D)**—t_24h_, (**E**–**H)**—t_24h+2h_. Primary antibodies: β-galactosidase monoclonal (1:50). Secondary antibodies: goat anti-mouse IgG DyLight^TM^488 (1:1000). Nuclear staining: Hoechst blue (1:1000). Magnification 20 × scale bar 10 µm.

**Figure 19:**
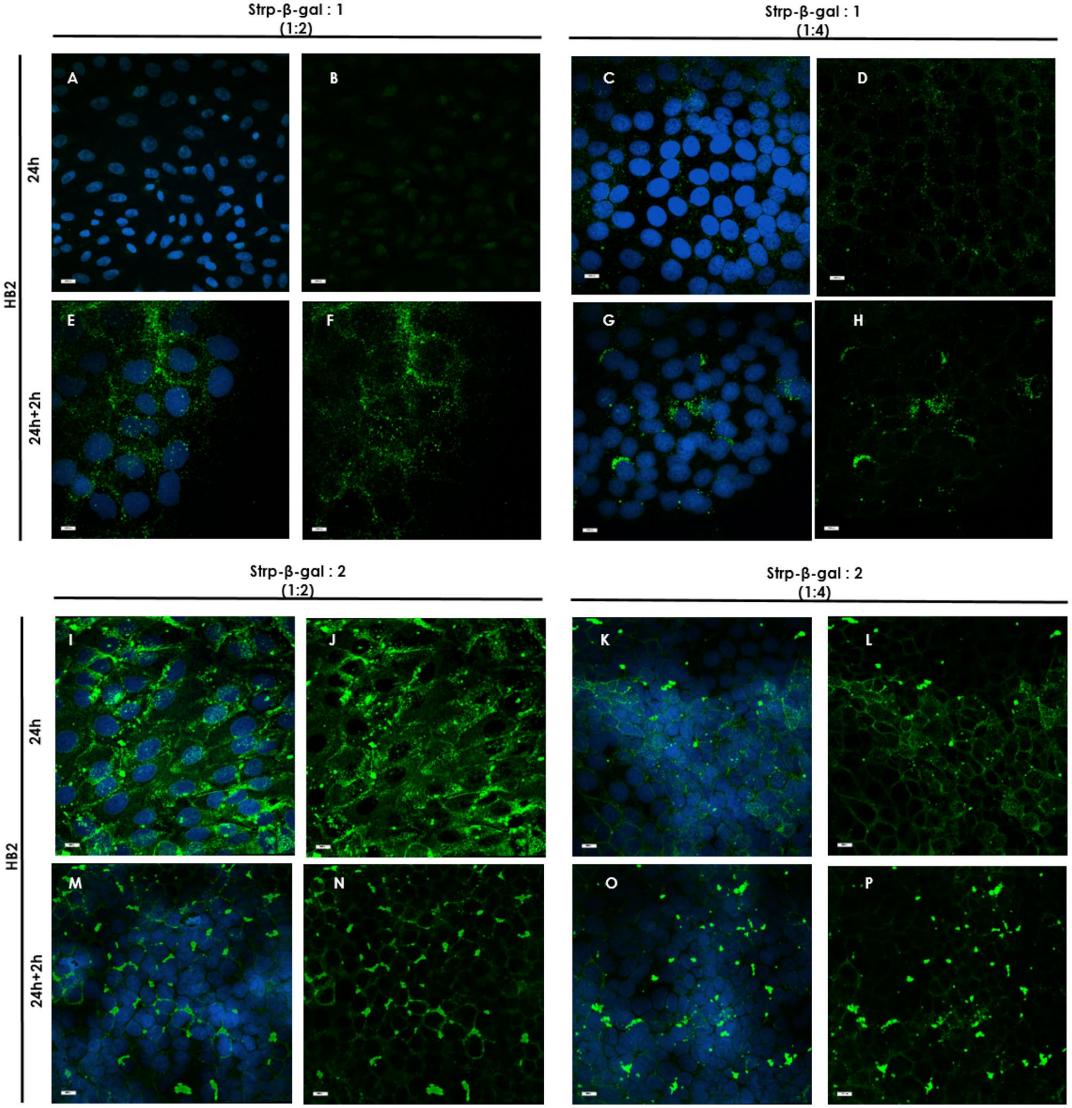
Immunofluorescence staining of HB2 cell cultures incubated with the following: (**A, B, E, F**)—Strp-β-gal:Compound 1 (molar ratio 1:2); (**C, D, G, H**)—Strp-β-gal:Compound 1 (molar ratio 1:4); (**I, J, M, N**)—Strp-β-gal:Compound 2 (molar ratio 1:2); (**K, L, O, P**)—Strp-β-gal:Compound 2 (molar ratio 1:4); (**A**–**D**) and (**I**–**L**)—t_24h_, (**E–H**) and (**M–P**)—t_24h+2h_. Primary antibodies: β-galactosidase monoclonal (1:50). Secondary antibodies: goat anti-mouse IgG DyLight^TM^488 (1:1000). Nuclear staining: Hoechst blue (1:1000). Magnification 20 × scale bar 10 µm.

**Figure 20:**
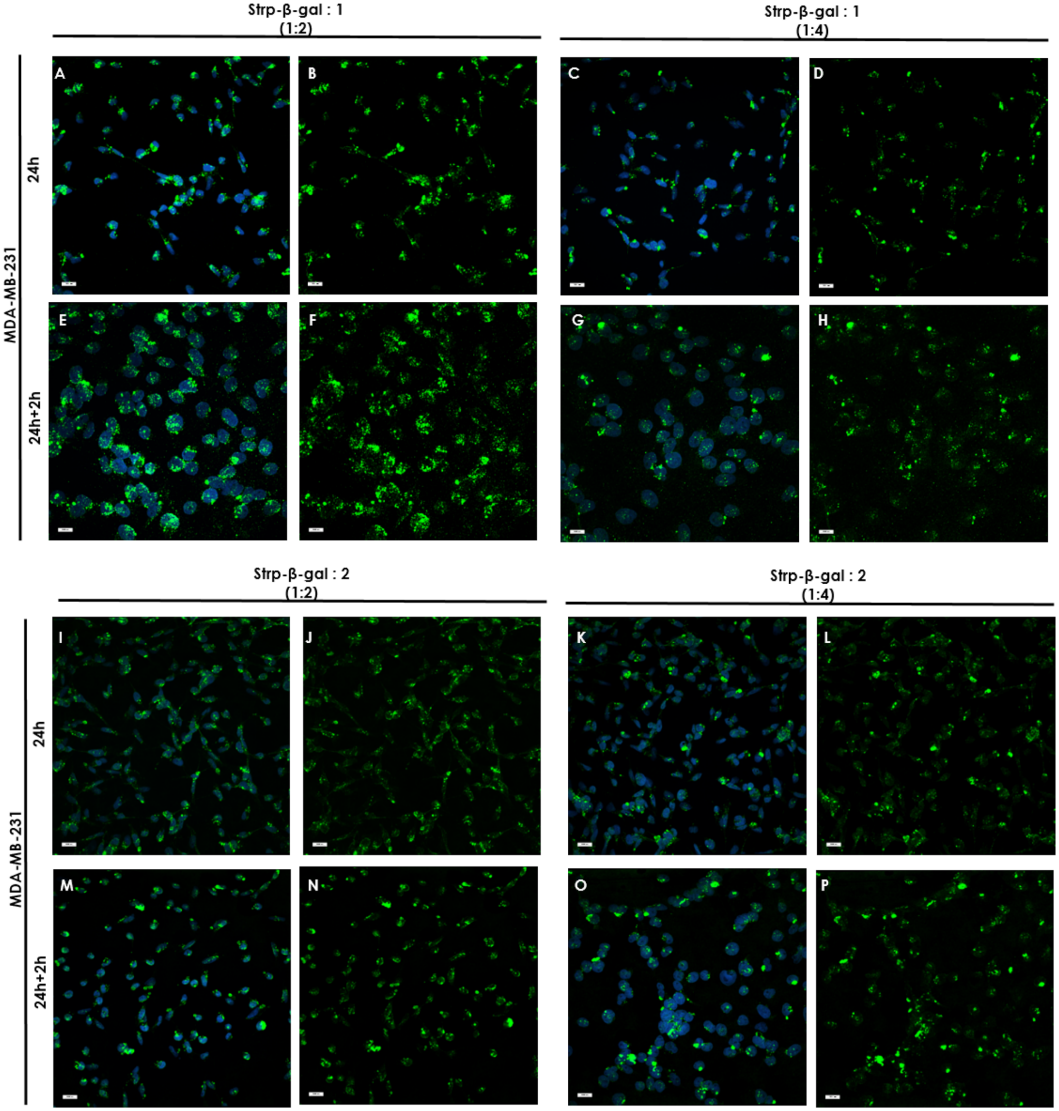
Immunofluorescence staining of MDA-MB-231 cell cultures incubated with (**A, B, E, F**)—Strp-β-gal: Compound 1 (molar ratio 1:2); (**C, D, G, H**)—Strp-β-gal: Compound 1 (molar ratio 1:4); (**I, J, M, N**)—Strp-β-gal: Compound 2 (molar ratio 1:2); (**K, L, O, P**)—Strp-β-gal: Compound 2 (molar ratio 1:4); (**A**–**D**) and (**I–L**)—t_24h_, (**E**–**H**) and (**M**–**P**)—t_24h+2h_. Primary antibodies: β-galactosidase monoclonal (1:50). Secondary antibodies: goat anti-mouse IgG DyLight^TM^488 (1:1000). Nuclear staining: Hoechst blue (1:1000). Magnification 20 × scale bar 10 µm.

To differentiate between endogenous β-gal activity, which can be found in every living cell at some background level, and the presence of the delivered enzyme, which is the active form of *E. coli*-expressed β-gal that is present as cargo in this experiment, we performed immunostaining experiments. As shown in Fig. 18, the control systems, cells treated with Strp-β-gal alone or without any additional component, display minimal fluorescence, which indicates the negligible presence of a secondary antibody labeled with DyLight^488^. Significantly elevated fluorescence was observed in all systems in which peptidomimetic Strp-β-gal conjugates were present (Figs. 19 and 20). Antibody staining was observed mainly in the membrane compartment but also in vesicle-like structures (Fig. 19b and c). The octamer mimetic was a more effective transporter of the Strp-β-gal complex than the hexamer regardless of the ratio used. Minimal differences between the two cell lines in terms of the amount of delivered enzyme were observed, with slightly more in the MDA-MB-231 cell lane.

**Figure 21:**
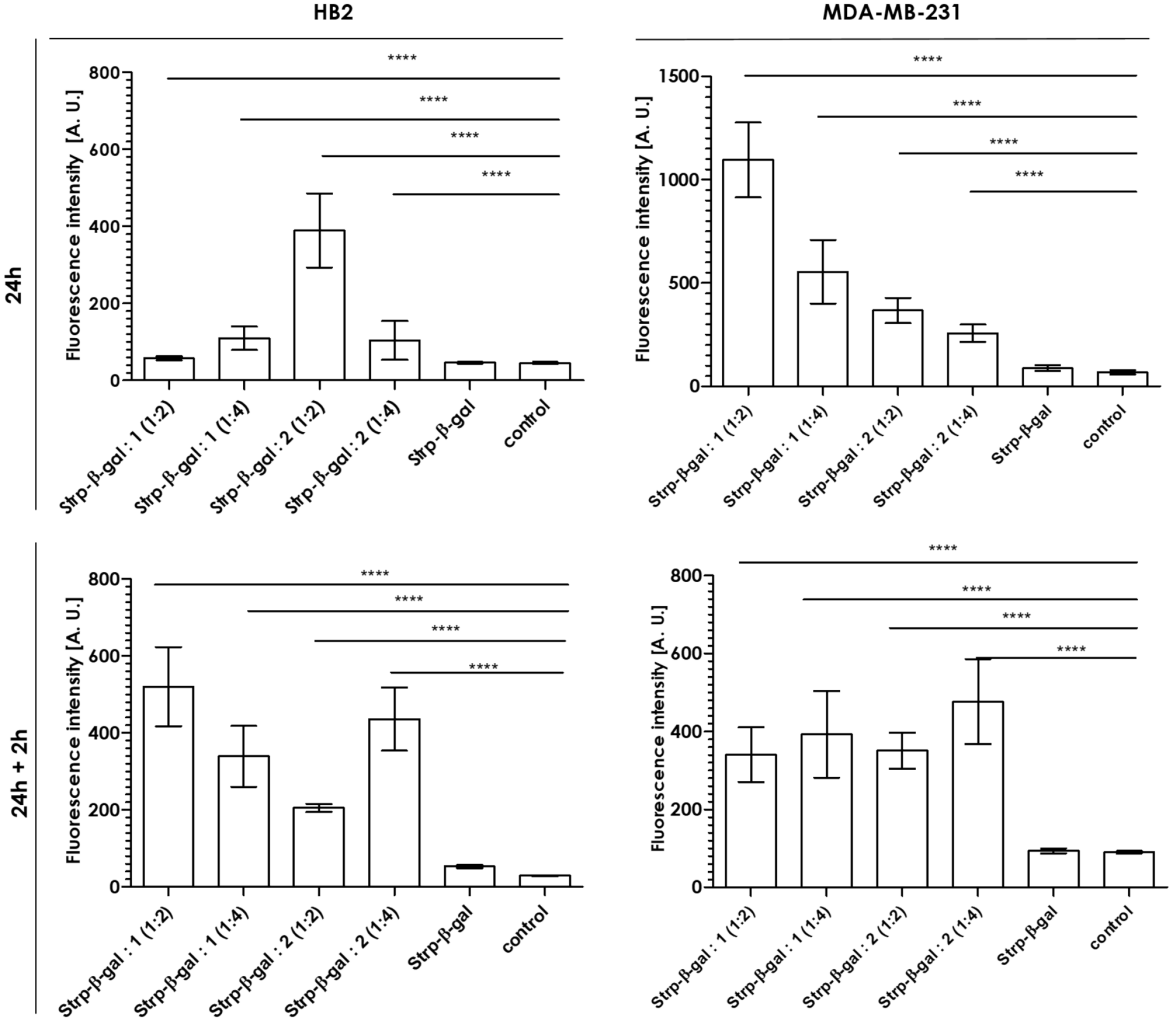
Mean fluorescence intensity of secondary antibody goat anti-mouse IgG DyLight^TM^488 conjugates in various systems.

There are several reports describing artificial translocation and subsequent localization of cationic compounds^22,23^. However, in light of all the above described experiments focusing on the localization and activity of the active enzyme form, we are in a position to state that this is not an issue in our case. Analyzing the overall fluorescence observed for particular well, we observed aa pattern that is presented in Fig. 21. Again, the presence of exogenous β-gal after 24 h of incubation with the complexes followed a similar pattern. For the HB2 cell lane, all systems, except the controls, displayed a greater amount of detected β-gal (Fig. 21). MDA-MB-231 cells were more sensitive to the composition and stoichiometry of the complexes used. In first time point (24 h) the greatest fluorescence was observed for compound 1 with dominance a stoichiometric ratio 1:2 system. The compound 2 was less effective in this moment. After additional two hours of incubation the all treated systems with both compounds displayed the same level of fluorescence that indicates effective transport into the cell interior. This observation is in parallel with enzyme activity (Fig. 14) Also microscopic observation recorded after additional 2 h, as visible in Fig. 19 (HB2 cells) and 20 (MDA-MB-231 cells), confirm this observations, the β-gal was mainly visible in vesicle-like structures. Regardless of the different stoichiometric ratios, the efficiency of β-gal in cell delivery remained constant. It is crucial to emphasize that the presence of β-gal as a protein detected by the antibody (Fig. 21) does not always correlate with its level of activity, as demonstrated in Fig. 14. In this particular instance, we observed an absence of direct correlation, but there was a discernible overall trend between the treated and non-treated systems.

In summary, the results presented above suggest the efficacy of a novel biotinylated cell-penetrating polymer able to mediate the cellular translocation of a model protein while retaining its activity. Such compounds could be utilized to transport functional exogenous or nonhost proteins into a variety of cells.

### Supplementary Information

Supplementary Figures.

## Data Availability

All publication-related data are included in the manuscript or supplementary materials.
