# Supplementary material for: Polymers of functionalized diaminopropionic acid are efficient mediators of active exogenous enzyme delivery into cells
Source: Sci Rep. 2024 Jun 8;14:13185. doi: 10.1038/s41598-024-64187-1 (PMC11162485; doi:10.1038/s41598-024-64187-1)
Supplement: Supplementary file 1 — Supplementary Figures. [file 41598_2024_64187_MOESM1_ESM.pdf]

Suplement materials

Title: Polymers of functionalized l-2,3-diaminopropionic acid are efficient mediators of active exogenous enzyme delivery into cells.

Authors:

Romanowska A.[1], Rachubik P.[2], Piwkowska A.[2], Wysocka M.[1#]

[1]Faculty of Chemistry, University of Gdansk, Wita Stwosza 63, 80-309 Gdansk, Poland

[2]Laboratory of Molecular and Cellular Nephrology, Mossakowski Medical Research Institute Polish Academy of Sciences, University of Gdansk, 80-308 Gdansk, Poland.

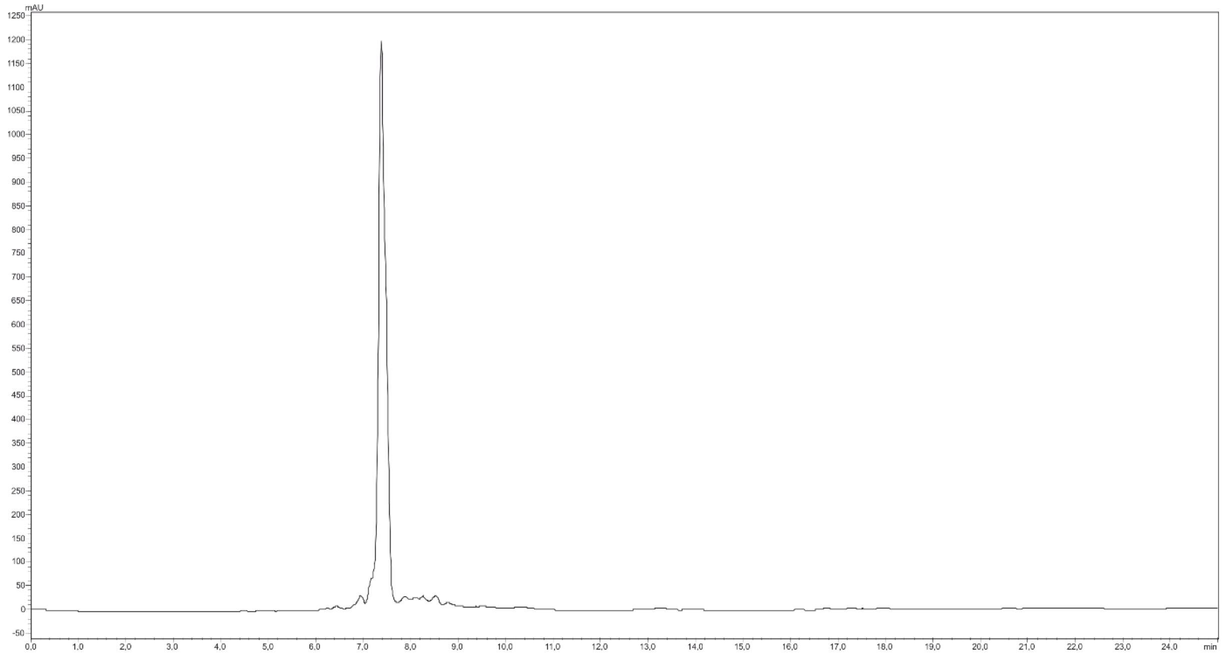

Fig 1S. UPLC analysis of compound 1. UPLC analysis (Nexera X2 LC-30AD [Shimadzu, Kyoto, Japan] equipped with a Phenomenex column (150 × 2.1 mm) with grain size 1.7 µm (peptide XB-C18) equipped with a UV–Vis detector and a fluorescence detector). Flow rate 0.3 ml/min. A linear gradient from 2% to 80% B within 15 min was applied (A: 0.1% trifluoroacetic acid; B: 80% acetonitrile in A).

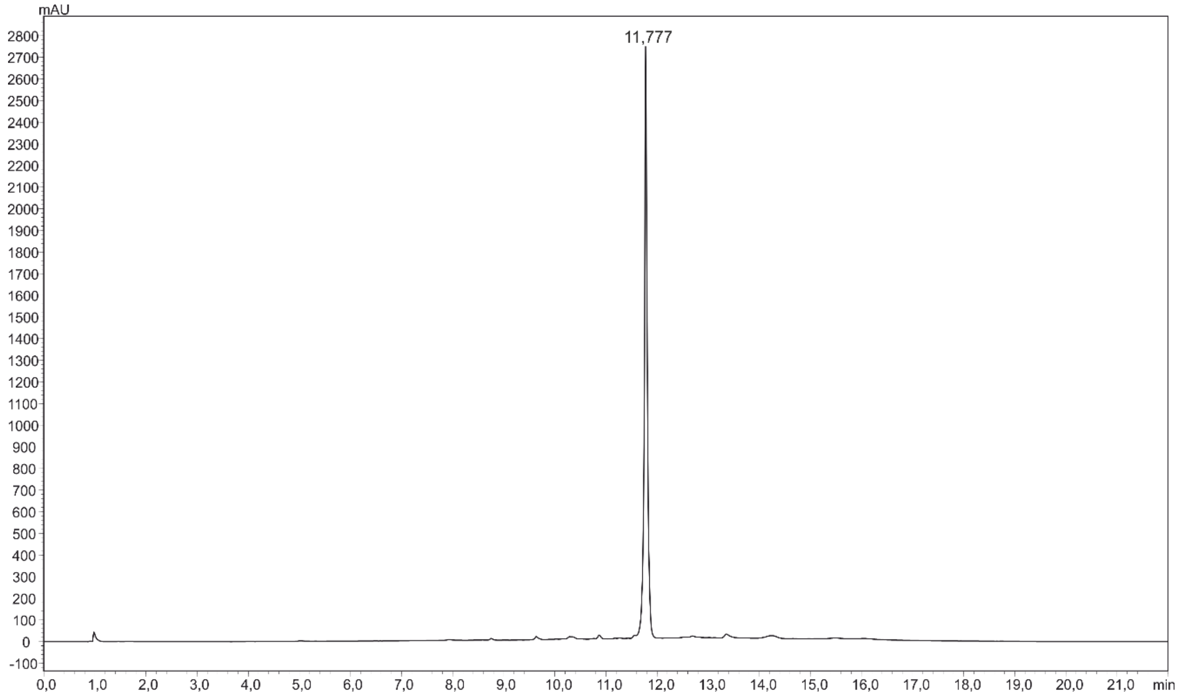

Fig 2S. UPLC analysis of compound 2. UPLC analysis (Nexera X2 LC-30AD [Shimadzu, Kyoto, Japan] equipped with a Phenomenex column (150 × 2.1 mm) with grain size 1.7 µm (peptide XB-C18) equipped with a UV–Vis detector and a fluorescence detector). Flow rate 0.3 ml/min. A linear gradient from 2% to 80% B within 15 min was applied (A: 0.1% trifluoroacetic acid; B: 80% acetonitrile in A).
